# Divalent cations and molecular crowding buffers stabilize G-triplex at physiologically relevant temperatures

**DOI:** 10.1038/srep09255

**Published:** 2015-03-19

**Authors:** Hong-Xin Jiang, Yunxi Cui, Ting Zhao, Hai-Wei Fu, Deepak Koirala, Jibin Abraham Punnoose, De-Ming Kong, Hanbin Mao

**Affiliations:** 1 State Key Laboratory of Medicinal Chemical Biology, Nankai University, Tianjin. 300071, P R China; 2 Collaborative Innovation Center of Chemical Science and Engineering (Tianjin), Tianjin. 300071, P R China; 3 Department of Chemistry & Biochemistry, Kent State University, Kent, OH 44242, USA

## Abstract

G-triplexes are non-canonical DNA structures formed by G-rich sequences with three G-tracts. Putative G-triplex-forming sequences are expected to be more prevalent than putative G-quadruplex-forming sequences. However, the research on G-triplexes is rare. In this work, the effects of molecular crowding and several physiologically important metal ions on the formation and stability of G-triplexes were examined using a combination of circular dichroism, thermodynamics, optical tweezers and calorimetry techniques. We determined that molecular crowding conditions and cations, such as Na^+^, K^+^, Mg^2+^ and Ca^2+^, promote the formation of G-triplexes and stabilize these structures. Of these four metal cations, Ca^2+^ has the strongest stabilizing effect, followed by K^+^, Mg^2+^, and Na^+^ in a decreasing order. The binding of K^+^ to G-triplexes is accompanied by exothermic heats, and the binding of Ca^2+^ with G-triplexes is characterized by endothermic heats. G-triplexes formed from two G-triad layers are not stable at physiological temperatures; however, G-triplexes formed from three G-triads exhibit melting temperatures higher than 37°C, especially under the molecular crowding conditions and in the presence of K^+^ or Ca^2+^. These observations imply that stable G-triplexes may be formed under physiological conditions.

The typical conformation of human genomic DNA is a double-stranded helix. However, some sequences can form non-canonical DNA structures, including G-quadruplex^1^, hairpin^2^, triplex^3^, Z-DNA^4^ and i-motif^5^. These structures have been associated with human diseases and, thus, are considered promising targets for therapeutic intervention^2^. The G-quadruplex is a well-known non-canonical DNA structure formed by G-rich DNA or RNA sequences (Figure 1) and has been studied extensively in recent years^1^. The basic unit of the structure is a G-tetrad, which is formed by four guanine bases situated in a plane and connected by Hoogsteen-type base pairing. Two or more G-tetrads stack on top of each other to form a G-quadruplex. The putative G-quadruplex-forming sequence motif, G_3+_N_1-7_G_3+_N_1-7_G_3+_N_1-7_G_3+_, occurs at more than 370,000 sites in the human genome^6^.

Intramolecular G-quadruplexes can form from a G-rich sequence containing four regions of two or more adjacent guanines (G-tracts), and these structures are stabilized by the monovalent K^+^ and Na^+^ ions. A G-rich sequence with only three G-tracts may form a G-triplex with stacked G-triads, in which a central guanine connects with two other guanines by Hoogsteen-like hydrogen-bonds (Figure 1)^7^,^8^,^9^,^10^. The formation of these G-triplexes has been hypothesized^11^,^12^,^13^, and experiments have suggested that they might be intermediates during folding or unfolding of G-quadruplexes^9^,^13^,^14^,^15^,^16^,^17^. Recently, Limongelli et al demonstrated the formation of G-triplexes in the G-rich sequence, 5'-GGTTGGTGTGG-3', using nuclear magnetic resonance (NMR) experiment in a dilute solution^7^,^8^. However, the melting temperature of the observed G-triplex was 33.5°C, suggesting that the structure is not stable at physiological temperatures. Given that intracellular environment is molecularly crowded, in which approximately 30~40% of the cellular volume can be occupied by macromolecules, here we wish to determine whether crowded buffers will promote the formation of G-triplexes and stabilize these structures, similar to those reported for G-quadruplex structures^18^,^19^,^20^,^21^,^22^,^23^.

Considering that the factors affecting the formation of G-triplexes have never been systematically studied, in this work, the effects of molecular crowding and several physiologically important metal ions on the formation and stability of G-triplexes were examined. We found that molecular crowding promotes the formation of G-triplex structures. We demonstrated that monovalent (K^+^ and Na^+^) and bivalent (Mg^2+^ and Ca^2+^) cations, particularly Ca^2+^, promote the formation of G-triplexes. This finding is different from that for G-quadruplexes, whose stabilities are almost unaffected by these two bivalent cations.

## Results and Discussion

### CD spectroscopy in dilute solutions

Six G-rich oligonucleotides were used to investigate the factors that influence G-triplex formation (Table 1). Three oligonucleotides (TBA, Hum21 and T_2_T_2_T_3_) with four G-tracts have the potential to form G-quadruplexes. The 3'-most G-tract of each of these oligomers was truncated to generate the TBA11, Hum15 and T_2_T_2_ fragments, which potentially form G-triplexes. As G-triplet and G-quartet share similar stacking and loop geometry, it is reasonable to assume that circular dichroism (CD) signals reflecting the strand orientation in the G-quadruplex may also apply to the G-triplex. We therefore used CD spectroscopy to examine the secondary structures of these six oligonucleotides in the presence of different metal ions, as well as under dilute and molecular crowding conditions. CD spectroscopy was first conducted under dilute conditions.

**Figure 2:**
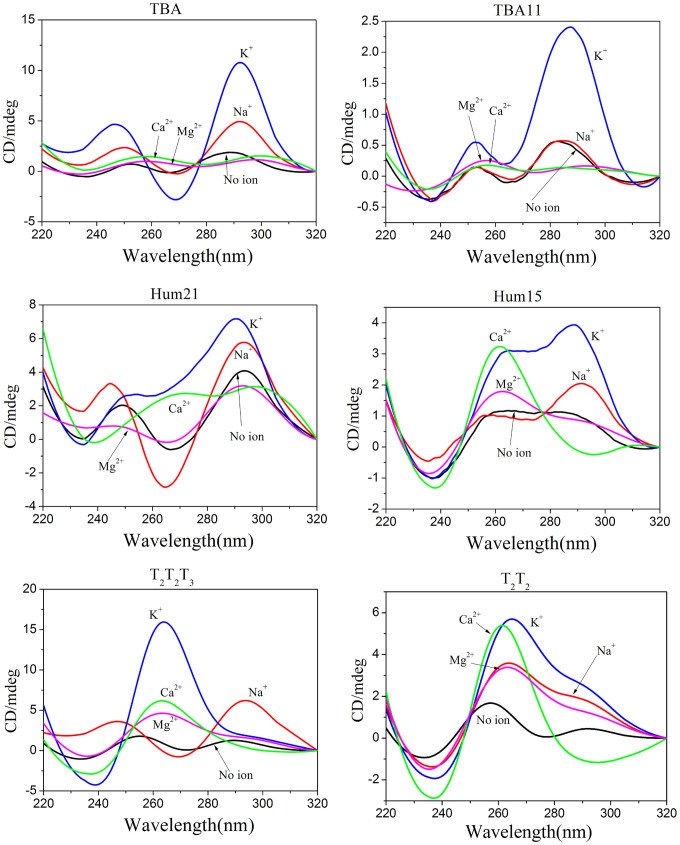
CD spectra of the six G-rich oligonucleotides in the presence of different metal ions under diluted conditions.

The thrombin-binding aptamer, TBA, is a well-known G-quadruplex-forming G-rich oligonucleotide that has been studied extensively. The truncated form of TBA, TBA11, has been shown to form a G-triplex structure by Limogelli and co-workers previously^7^,^8^. The CD spectra of TBA in a dilute solution containing K^+^ or Na^+^ had a positive peak at approximately 292 nm and a negative peak at approximately 269 nm (Figure 2). These results are characteristic of antiparallel G-quadruplexes^24^, indicating that K^+^ and Na^+^ promote antiparallel G-quadruplex formation of TBA. Under the same conditions, TBA11 in presence of K^+^ revealed a similar CD spectrum profile with a positive peak around 288 and a negative peak around 264 nm, indicating the formation of antiparallel G-triplex by TBA11. These features are identical to those reported by Limogelli et al in which G-triplex formation was confirmed by NMR investigations^7^,^8^. However, it should be noted that the CD signal intensity of the TBA11 G-triplex was much lower than that of the TBA G-quadruplex, suggesting the probability of G-triplex formation is less than that of G-quadruplex. Under these dilute conditions, neither TBA nor TBA11 showed obvious CD signals in the presence of Ca^2+^ or Mg^2+^.

Next, we investigated human telomeric sequences, Hum21 and Hum15 (Figure 2). Under the dilute conditions without metal ions, the positive peak at around 294 nm and the negative peak at around 266 nm in the CD spectrum of Hum21 indicated partial formation of antiparallel G-quadruplexes. The presence of K^+^ or Na^+^ further promoted the formation of G-quadruplex structures. In contrast to TBA, Hum21 exhibited antiparallel G-quadruplex structure in the presence of Na^+^ and parallel/antiparallel-mixed hybrid G-quadruplex structure in the presence of K^+^ ion^25^. Both K^+^ and Na^+^ promoted G-triplex formation by Hum15 and the effect of K^+^ was notably stronger than that of Na^+^. In the presence of K^+^ or Na^+^, Hum15 appeared to form a parallel/antiparallel hybrid G-triplex, as indicated by a positive CD peak at approximately 290 nm, a shoulder peak at approximately 265 nm and a negative CD peak at approximately 235 nm.

The features of CD spectrum in the Ca^2+^ buffer are rather different from those in K^+^ and Na^+^ buffers. This may be due to different strand orientations in the G-triplex. In the presence of Ca^2+^, the CD spectrum of Hum15 had a strong positive peak at approximately 262 nm and a negative peak at 236 nm, which are typical for parallel strand arrangement^24^, Under the same conditions, the CD spectrum of Hum21 in the presence of Ca^2+^ had a negative peak at approximately 236 nm and two weak positive peaks at approximately 268 nm and 297 nm. It is possible that a mixture of two G-quadruplex structures was present in the solution; however, the possibility of a mixture of G-triplexes and G-quadruplexes cannot be excluded. Mg^2+^ did not notably promote G-quadruplex formation of Hum21. However, it seemed that Mg^2+^ promoted the formation of G-triplexes, as evidenced by a positive peak at approximately 263 nm in the Hum15 CD spectrum.

Under dilute conditions, T_2_T_2_T_3_ formed parallel or antiparallel G-quadruplex structures in the presence of K^+^ or Na^+^, respectively (Figure 2). However, the presence of either K^+^ or Na^+^ promoted the formation of hybrid G-triplex structures of T_2_T_2_. The hybrid structure differed from those formed by Hum15 in the presence of K^+^, as CD peaks for T_2_T_2_ in the presence of K^+^ or Na^+^ were stronger at 266 nm than at 294 nm. In contrast, CD peaks for Hum15 in the presence of K^+^ or Na^+^ were stronger at 294 nm than at 266 nm. In the presence of Ca^2+^ or Mg^2+^, T_2_T_2_ showed similar CD spectrum profiles to Hum15, suggesting they share similar G-triplex conformations.

**Figure 3:**
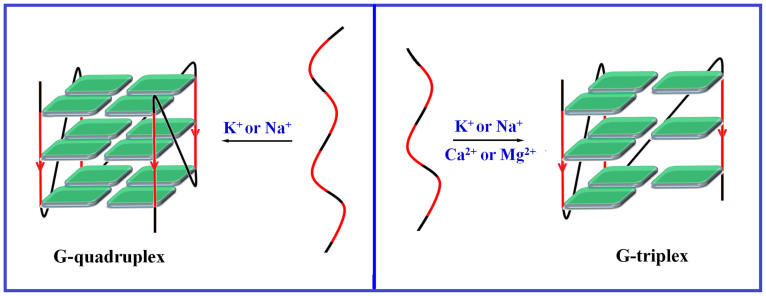
K^+^ and Na^+ ^promote the formation of G-quadruplexes; while K^+^, Na^+^, Ca^2+^ and Mg^2+^ promote the formation of G-triplexes.

Overall, the presence of Na^+^, K^+^, Ca^2+^ or Mg^2+^ under dilute conditions promoted the formation of G-triplexes to some degree (Figure 3). However, the effects of K^+^ and Ca^2+^ were much stronger than those of Na^+^ and Mg^2+^. In addition, K^+^ and Ca^2+^ affected G-triplex formation differently. It seems that G-rich sequences tend to form parallel G-triplexes in the presence of Ca^2+^. However, G-triplexes formed in the presence of K^+^ may have different sequence-dependent conformations. It is possible that G-triplexes formed under this condition may have a strand orientation similar to corresponding G-quadruplexes (Table S1). In contrast to the well-known promotion of the formation and stabilization of G-quadruplexes by K^+^ and Na^+^, the presence of Ca^2+^ or Mg^2+^ promotes G-quadruplex formation weakly at best. Importantly, Ca^2+^ and Mg^2+^ selectively promote the formation of G-triplexes.

### CD spectroscopy under molecular crowding conditions

**Figure 4:**
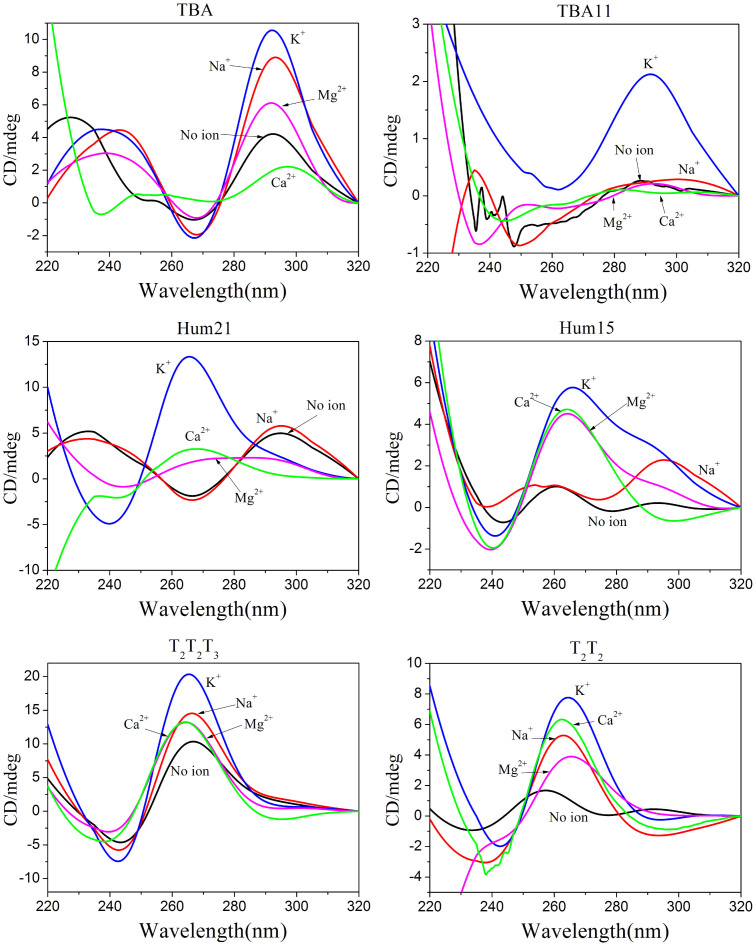
CD spectra of the six G-rich oligonucleotides in the presence of different metal ions under molecularly crowded conditions.

It has been reported that molecular crowding can affect the conformation and stability of G-quadruplexes^18^,^19^,^20^,^21^,^22^,^23^. We used polyethylene glycol 200 (PEG 200) as a molecular crowding agent to investigate the formation of G-quadruplexes or G-triplexes in the presence of different metal ions (Figure 4)^26^. In the presence of 40% (v/v) PEG 200, K^+^ promoted the formation of G-triplexes in the TBA11, while Na^+^, K^+^, Ca^2+^ and Mg^2+^ promoted G-triplex formation in the Hum15 and T_2_T_2_ fragments. As observed for dilute conditions, G-triplexes formed in the Hum15 and T_2_T_2_ may assume parallel strand orientation in the presence of Ca^2+^ or Mg^2+^ under molecular crowding conditions. The G-triplexes formed in the presence of K^+^ or Na^+^ exhibited similar strand orientations to the G-quadruplexes formed in the corresponding longer oligonucleotides. Similar to G-quadruplex, we found molecular crowding promoted parallel G-triplex formation for some oligonucleotides. For example, in the presence of K^+^, T_2_T_2_ showed a potential G-triplex structure with strand orientations similar to the hybrid G-quadruplex in a dilute buffer. However, the structure more likely assumes a parallel strand orientation under the molecular crowding conditions (Figure 4).

### CD spectra of four mutants lacking the potential to form G-triplexes

**Figure 5:**
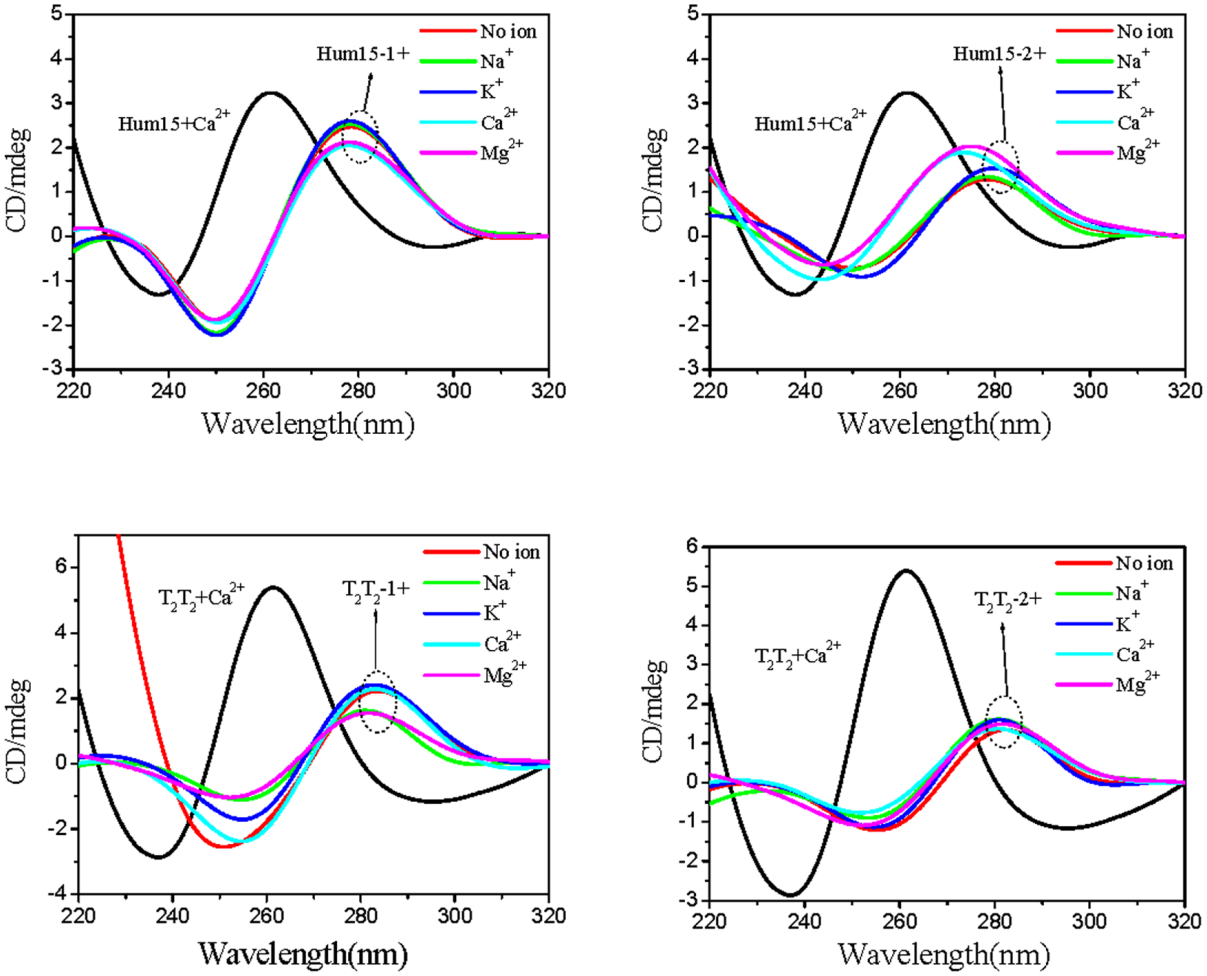
CD spectra of the four mutants lacking G-triplex-forming abilities in the absence or presence of different metal ions under diluted conditions.

**Figure 6:**
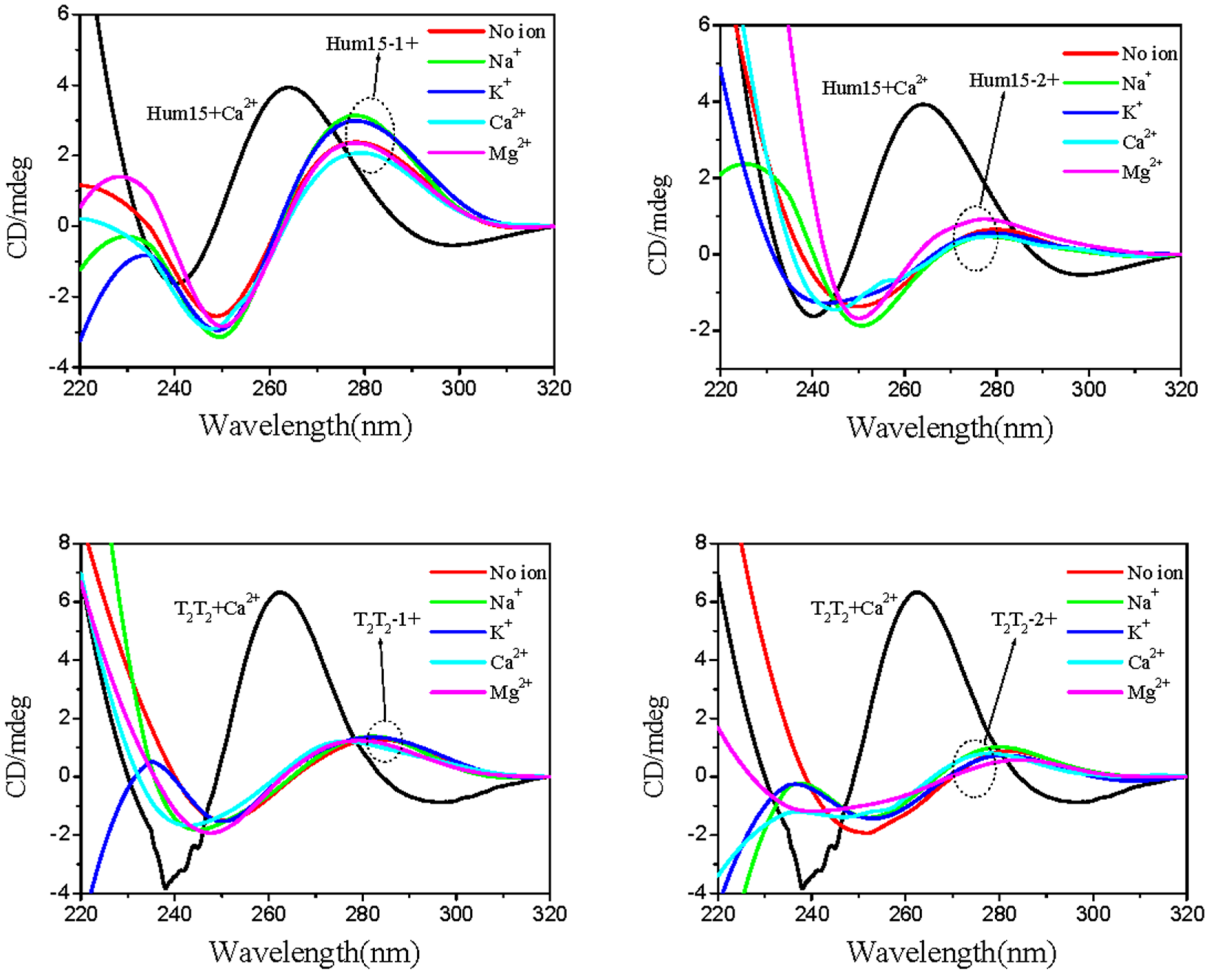
CD spectra of the four mutants lacking G-triplex-forming abilities in the absence or presence of different metal ions under molecularly crowded conditions.

To support that observed CD spectra reflect the formation of G-triplexes, four mutants, Hum15-1, Hum15-2, T_2_T_2_-1 and T_2_T_2_-2 (Table 1), were designed by replacing G residues essential for the G-triplex formation with nucleotide substitutions (C or T). As expected, the four mutant oligonucleotides exhibited the same CD spectra under either diluted or crowded buffers, regardless of the presence of metal ions (Na^+^, K^+^, Mg^2+^ or Ca^2+^) (Figure 5 and Figure 6). The observed positive peaks at approximately 280 nm were characteristic of single-stranded or double-stranded DNA^27^. These results support that the ion- or molecular-crowding-induced changes in CD signals shown in Figures 2 and 4 were not caused by simple interactions between the metal ions and the nucleotide bases. Instead, they could be the result of G-triplex formation.

### Melting temperature (*T*_m_) assay was used to demonstrate the formation of G-triplexes

G-rich sequences with fewer than four G-tracts might possibly also form intermolecular G-quadruplexes^28^,^29^. Thus, the DNA secondary structures formed by TBA11, Hum15 and T_2_T_2_ might be intermolecular G-quadruplexes rather than G-triplexes. It has been reported that the stability will increase with DNA concentration for intermolecular structures but not for intramolecular structures^30^,^31^. To demonstrate the intramolecular G-triplexes formation by these G-rich oligonucleotides, DNA-concentration dependent stability change of the proposed G-triplexes was investigated. The stability change can be reflected by the melting temperature (*T*_m_) of the DNA secondary structures. Similar to G-quadruplexes^32^, DNA secondary structures formed in the TBA11, Hum15, or T_2_T_2_ exhibited decreased UV absorption at 295 nm with increasing temperature (Figure S1). In contrast, the oligonucleotides without G-quadruplex and G-triplex-forming potential did not exhibit similar behaviour (Figure S2). These experiments support that stable structures, likely those with a stack of G-triads as suggested from CD experiments, exist in TBA11, Hum15, or T_2_T_2_ fragments. These results also imply that, as with G-quadruplexes, the temperature-dependent changes in 295 nm UV absorbance can be used to determine the *T*_m_ of the G-triplex structures.

To exclude the possibility of intermolecular G-quadruplex formation, *T*_m_ changes of the three G-rich oligonucleotides (TBA, Hum15, T_2_T_2_) were determined with different DNA concentrations (7–15 μM). The results indicate that the *T*_m_ values were independent of DNA concentration in the presence of individual metal ions and under both diluted and crowded buffers (Figure S3–S5, Table S2), confirming that these three oligonucleotides form intramolecular structures, i.e., G-triplexes.

The G-rich Hum9 oligonucleotide (Table 1), a truncated human telomeric sequence, was used to further exclude the possibility of intermolecular G-quadruplex formation. Because it has only two GGG repeats, it can form only intermolecular G-quadruplex structures. The result of CD spectroscopy indicated that only the CD spectra under K^+^ condition differed from the control without ions (Figure S6), indicating that only K^+^ promoted the formation of intermolecular G-quadruplexes (i.e., Na^+^, Mg^2+^, or Ca^2+^ did not). This result supports that the DNA secondary structures formed by Hum15 and T_2_T_2_, especially in the presence of Na^+^, Mg^2+^ or Ca^2+^, were intramolecular G-triplexes and not intermolecular G-quadruplexes. Melting temperature analysis indicated that the intermolecular G-quadruplexes formed by Hum9 were less stable than the proposed G-triplexes formed by Hum15 in the presence of K^+^, thus suggesting that Hum15 may preferentially form more stable intramolecular G-triplexes in the presence of K^+^.

### Stabilization of G-quadruplexes and G-triplexes by metal ions and molecular crowding conditions

Having demonstrated the promoting effect of Na^+^, K^+^, Ca^2+^ and Mg^2+^ on the formation of G-triplexes under both dilute and molecular crowding conditions, the effects of these ions on G-triplex stability were compared (Figure S1). As summarized in Table 2, under both diluted and crowded conditions, the triplex-stabilizing effect of K^+^ was stronger than Na^+^. This result corresponds well with their effects on G-quadruplex stability. G-quadruplexes formed in the presence of Ca^2+^ exhibited lower *T*_m_ values than those formed in the presence of K^+^, indicating that the G-quadruplex-stabilizing effect of K^+^ is better than that of Ca^2+^ ion^32^. In contrast, Ca^2+^ exhibited stronger G-triplex-stabilizing effects than Na^+^, K^+^ or Mg^2+^. The strength of G-triplex-stabilizing effects was ranked as Ca^2+^>K^+^>Mg^2+^>Na^+^. In the presence of Na^+^, K^+^, Mg^2+^ or Ca^2+^, G-triplexes formed in crowded buffers exhibited higher *T*_m_ values than those formed in dilute solutions, indicating that molecular crowding stabilizes G-triplexes. Similar results were obtained for G-quadruplex studies^19^.

In the presence of Na^+^ or K^+^, G-triplexes exhibited lower *T*_m_ values than corresponding G-quadruplexes, suggesting that G-quadruplexes are more stable than the corresponding G-triplexes.

Although the G-quadruplex consisting of two G-tetrads (TBA) was relatively stable in the presence of K^+^ (*T*_m_ of 51.1°C under the molecular crowding condition), the G-triplex consisting of two G-triads (TBA11) was much less stable (*T*_m_ of 34.3°C in the presence of Ca^2+^ and under the molecular crowding condition). This observation may indicate that G-triplexes with two G-triads might not be stable at physiological temperatures. In contrast, the G-triplexes with three G-triads are much more stable, especially in the presence of K^+^ or Ca^2+^. Under the molecular crowding conditions, the *T*_m_ values of Ca^2+^-stabilized G-triplexes (Hum15 and T_2_T_2_) were 52.0°C and 64.5°C, respectively. With K^+^ or Ca^2+^ stabilization, G-triplex structures with three G-triads might be stable at physiological temperatures.

### Thermodynamic profiles for the formation of G-triplexes and G-quadruplexes

**Figure 7:**
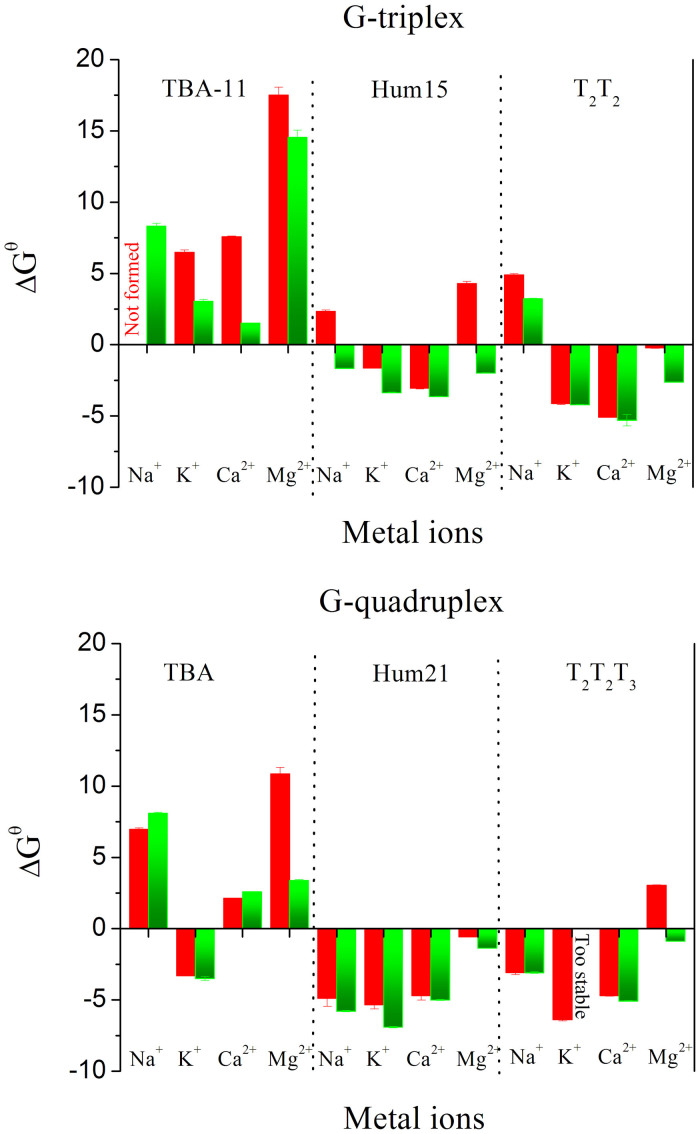
Free energy change at 37°C (ΔG^θ^_37_) during G-triplex and G-quadruplex formation under different conditions. (Red bars) under dilute conditions; (Green bars) under molecularly crowded conditions.

The *T*_m_ provides a rough measurement for the stability of a structure at a specific temperature. To quantify thermodynamic stabilities, we used a two-state transition model to evaluate the free energy change at 37°C (Δ*G*^θ^_37_) of these G-rich sequences by analyzing the melting curves (Figure 7, Table S3)^30^. Under both dilute and molecular crowding conditions, TBA11 gave positive Δ*G*^θ^_37_ values in the presence of either metal ion, confirming that G-triplexes containing two G-triads cannot be formed at physiological temperatures. However, negative Δ*G*^θ^_37_ values were found in Hum15 and T_2_T_2_, especially in the presence of K^+^ or Ca^2+^, indicating that stable G-triplexes can be formed at physiological temperatures in the presence of these two ions. The favourable Δ*G*^θ^_37_ is a result of the characteristic compensation of a favourable enthalpy term with an unfavourable entropy term (Table S3)^33^. In the presence of K^+^, the Δ*G*^θ^_37_ values of G-quadruplexes are more favourable than those of corresponding G-triplexes, and the G-quadruplexes stabilized by K^+^ generate more favourable Δ*G*^θ^_37_ values compared to those stabilized by Ca^2+^. These results demonstrate that K^+^ is a better G-quadruplex stabilizer than Ca^2+^. In the presence of Ca^2+^, G-triplexes yield ΔG^θ^_37_ values comparable to or even more favourable than G-quadruplexes. In addition, the G-triplexes stabilized by Ca^2+^ have more favourable ΔG^θ^_37_ values than those stabilized by K^+^. These results suggest that Ca^2+^ is a better G-triplex stabilizer than K^+^, which is opposite to the scenario in G-quadruplex.

### Binding affinities of K^+^ or Ca^2+^ to G-quadruplexes or G-triplexes

**Figure 8:**
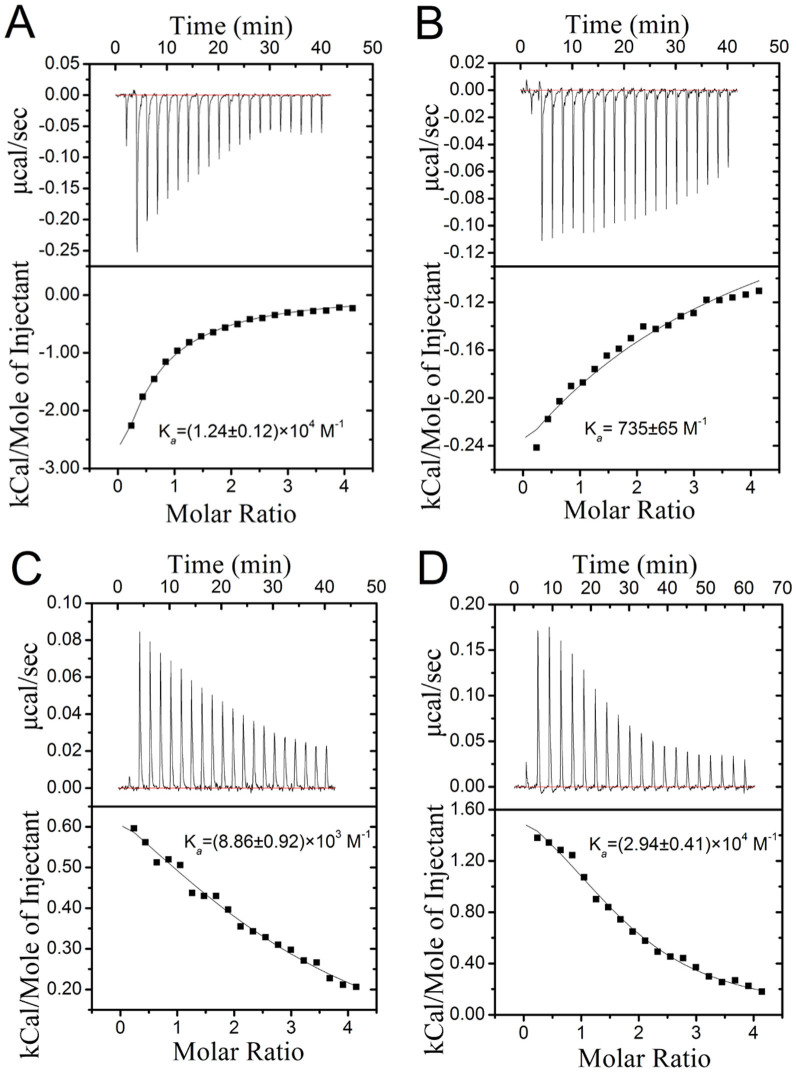
Calorimetric curves and binding constant between K^+^ or Ca^2+^ and Hum21 or Hum15 measured by ITC. (A) titration of Hum21 with K^+^. (B) titration of Hum15 with K^+^. (C) titration of Hum21 with Ca^2+^. (D) titration of Hum15 with Ca^2+^.

Isothermal titration calorimetry (ITC) is a quantitative technique that can determine the binding affinity (*K*_a_) of the interaction between two or more molecules in solution. This technique has been used to study the binding constant between G-quadruplexes and metal ions or ligands^34^,^35^. Herein, the interactions of K^+^ and Ca^2+^ with G-quadruplexes or G-triplexes were also investigated using ITC assays. As shown in Figure 8, the binding of K^+^ to Hum21 and Hum15 is accompanied by exothermic heats. K^+^ binds the G-triplex Hum15 with a *K*_a_ value of 735 ± 65 M^−1^, which is ~17-fold weaker than that of K^+^ and the G-quadruplex Hum21 (*K*_a_ = (1.24 ± 0.12) × 10^4^ M^−1^). This is consistent with the above observation that K^+^ has higher stabilizing ability to G-quadruplexes than to G-triplexes. The binding of Ca^2+^ with G-quadruplexes or G-triplexes is characterized by endothermic heats. In contrast to K^+^, Ca^2+^ shows a higher binding affinity to G-triplex (*K*_a_ = (2.94 ± 0.41) × 10^4^ M^−1^) than to G-quadruplex (*K*_a_ = (8.86 ± 0.92) × 10^3^ M^−1^). And as expected, Ca^2+^ shows a higher binding affinity to G-triplex than K^+^. These results are also consistent those of CD and melting assays, thus further demonstrating that Ca^2+^ is a better G-triplex stabilizer than K^+^.

### Ca^2+^ concentration-dependent *T*_m_ change of Hum15

**Figure 9:**
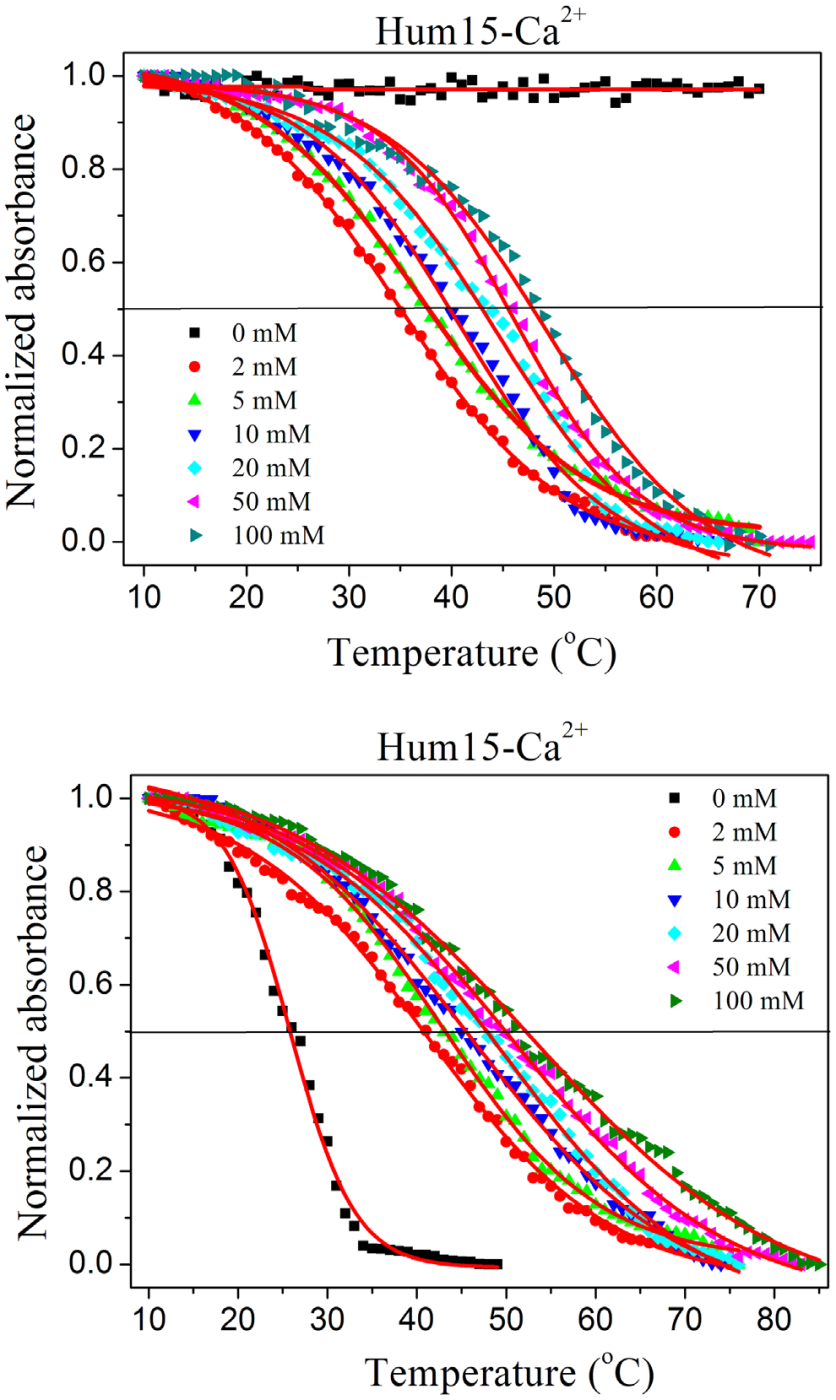
Ca^2+^ concentration-dependent melting temperature change of the G-triplex formed by Hum15 under diluted (Top) or molecularly crowded conditions (Bottom).

As Ca^2+^ exerted the strongest G-triplex-promoting and stabilizing effects, the effect of Ca^2+^ concentration on the *T*_m_ of the Hum15 G-triplex was investigated. As shown in Figure 9, under the dilute condition, temperature-dependent absorption signal change was not observed in the absence of Ca^2+^, indicating that Hum15 cannot form G-triplexes without Ca^2+^. G-triplex formation was promoted by the addition of 2 mM Ca^2+^, and the resulting G-triplex exhibited a *T*_m_ of 35.0°C. The *T*_m_ of the G-triplex increased as the Ca^2+^ concentration was increased from 2 mM to 100 mM (Table S4). Under the molecular crowding conditions, even in the absence of Ca^2+^, Hum15 can form some G-triplexes which have a *T*_m_ of 25.9°C. Increment of *T*_m_ with Ca^2+^ concentration was also observed in crowded buffers. These results indicate that Ca^2+^ indeed stabilizes G-triplexes under both diluted and molecularly crowded conditions.

Inside cells, K^+^ has a concentration of ~140 mM, Na^+^ has a concentration of ~10 mM, whereas both Ca^2+^ and Mg^2+^ have significant concentrations of μM-mM^36^,^37^, especially under certain biological activities. Therefore, it is of high physiological significance that these cations would promote the formation of the G-triplexes and stabilize these structures. With the finding that molecular crowding also favours the G-triplex formation, it is possible that like G-quadruplex, G-triplex may exist in vivo for potential biological functions.

### The promotion effect of Ca^2+^ to G-triplex formation is confirmed at the single-molecular level

**Figure 10:**
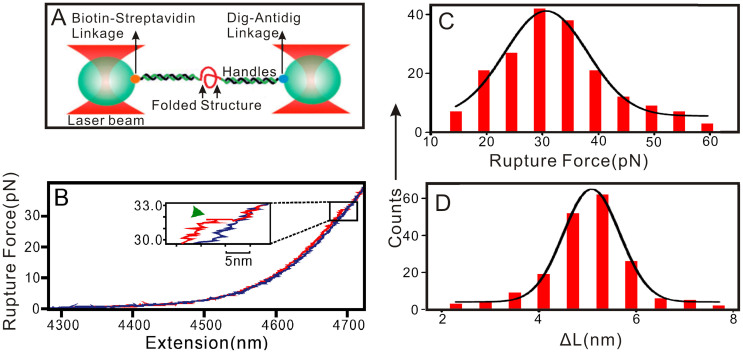
Mechanical unfolding of Hum15 in the presence of 100 mM Ca^2+^. (A) Schematic of the single-molecule mechanical unfolding assay (not to scale). (B) A typical force-extension (F-X) curve for Hum15. The red and blue depict the stretching and relaxing Hum15 curves, respectively. The green arrowhead shows the unfolding event. Change in contour length (Δ*L*) (C) and rupture force (D) histograms for structures in Hum15. The solid curves in (C) and (D) are Gaussian fittings.

Optical tweezers represent a unique approach to investigate the mechanical stability of folded nucleic acid structures at the single-molecule level^14^,^38^,^39^,^40^,^41^. This technique has been used to demonstrate the formation of G-triplex structures formed by human telomeric sequences containing three GGG repeats under dilute conditions and in the presence of Na^+^ ion^14^. To further demonstrate the effect of Ca^2+^ on the G-triplex formation, optical tweezers assay was conducted to unfold structures in the Hum15 under three conditions (100 mM K^+^, 100 mM Ca^2+^, and 100 mM K^+^ + 2 mM Ca^2+^). When single-stranded Hum15 tethered between two duplex handles was stretched, a characteristic unfolding event was observed from the force-extension curve, indicating that folded structure was formed in the Hum15 (Figure 10 and Figure S7–S10). In the presence of 100 mM Ca^2+^, this structure could be disrupted at the rupture force of 31 ± 3 pN, with the change in contour length (Δ*L*) of 5.1 ± 0.5 nm. This Δ*L* value matches very well with that of G-triplex^14^, thus strongly supporting that Hum15 folds into G-triplex structure. The possibility of G-triplex formation increased from 21 ± 2% under 100 mM K^+^ to 48 ± 3% under 100 mM Ca^2+^, indicating that the capability of Ca^2+^ to promote G-triplex formation is better than that of K^+^. The G-triplex-promoting ability of Ca^2+^ could also be observed when Ca^2+^ has a low concentration. For example, in the presence of 100 mM K^+^ + 2 mM Ca^2+^, 32 ± 2% Hum15 could fold into G-triplexes.

Since stable optical trapping requires a significant difference in the refractive index of a trapped particle and solvent, we used 40% dimethyl sulfoxide (DMSO) to simulate molecular crowding conditions^23^. The mechanical unfolding results showed that molecular crowding conditions indeed could promote the formation of G-triplexes. In the presence of 40% DMSO, the possibility of G-triplex formation increased from 21 ± 2% to 29 ± 2% under 100 mM K^+^, and from 32 ± 2% to 35 ± 3% in the presence of 100 mM K^+^ + 2 mM Ca^2+^.

Interestingly, the rupture forces under these conditions are similarly located at 31–35 pN (Figure 10, Figure S7–S10). These values are higher than the stall force of many RNA polymerase^42^, which suggests that the G-triplexes may serve as a mechanical block for transcription processes.

## Conclusion

In summary, we demonstrated that G-triplex structures can be formed by G-rich sequences containing only three G-tracts. In addition, molecular crowding and the presence of physiological important metal ions, such as Na^+^, K^+^, Ca^2+^ and Mg^2+^, promote and stabilize G-triplex formation. The strength of the G-triplex-stabilizing effects of these ions is found to be: Ca^2+^>K^+^>Mg^2+^>Na^+^. Ca^2+^ promotes the formation of parallel G-triplexes under either dilute or molecular crowding conditions. G-triplexes formed in the presence of K^+^ often have strand orientations similar to those of G-quadruplexes formed in the corresponding oligonucleotides containing four G-tracts. The melting temperatures of the G-triplexes consisting of two G-triads are lower than physiological temperature, even under the molecular crowding condition and in the presence of Ca^2+^. However, the G-triplexes containing three G-triads have much higher melting temperatures, especially under the molecular crowding condition and in the presence of K^+^ or Ca^2+^, implying that stable G-triplexes may be formed at physiological temperatures. The binding of K^+^ to G-triplexes is accompanied by exothermic heats, and the binding of Ca^2+^ with G-triplexes is characterized by endothermic heats. The G-triplex-promoting and stabilizing effect of Ca^2+^ is noteworthy, as Ca^2+^ is a physiologically important metal ion and it exhibits rather weak or undetectable effect on the G-quadruplex structures. As G-quadruplexes are believed to have important biological function and the number of putative G-triplex-forming sequences might be more prevalent in the human genome than putative G-quadruplex-forming sequences, our study may pave a way for further studies on the G-triplexes, such as whether G-triplexes form in vivo and whether they have biological functions.

## Methods

### Materials and reagents

The oligonucleotides listed in Table 1 were purchased from Sangon Biotech. Co. Ltd (Shanghai, China). The concentrations of the oligonucleotides were represented as single-stranded concentrations. Single-stranded concentration was determined by measuring the absorbance at 260 nm. Molar extinction coefficient was determined using a nearest neighbour approximation (http://www.idtdna.com/analyzer/Applications/OligoAnalyzer). Na_2_EDTA (Disodium ethylenediamine tetraacetic acid), Tris [tris(hydroxymethyl)aminomethane], KCl, NaCl, MgCl_2_, CaCl_2_, PEG 200, HCl were obtained from Sigma. Deionized and sterilized water (resistance > 18 MΩ/cm) was used throughout the experiments.

### Circular dichroism (CD) spectroscopy

Under dilute condition, 3 mL reaction mixture was prepared in 10 mM Tris–HCl buffer (pH = 7.0) containing 2.5 μM individual DNA oligonucleotides, 0.5 mM Na_2_EDTA, and 100 mM individual metal ions. Under molecular crowding conditions, 3 mL reaction mixture was prepared in 10 mM Tris–HCl buffer (pH = 7.0) containing 2.5 μM individual DNA oligonucleotides, 0.5 mM Na_2_EDTA, 400 mL/L PEG 200, and 100 mM individual metal ions. The mixture was heated at 95°C for 5 min, cooled to 25°C and then incubated at 4°C overnight. CD spectrum of the mixture was recorded between 230 and 320 nm in 1-mm path length cuvettes on a Jasco J-715 spectropolarimeter. Spectra were averaged from three scans, which were recorded at 100 nm/min with a response time of 1 s and a bandwith of 1.0 nm.

### Melting temperature detection of G-quadruplexes or G-triplexes

Melting temperature detection of G-quadruplexes or G-triplexes was carried out on a Cary-60 UV-vis spectrophotometer equipped with a single cell peltier temperature control accessory. Under dilute condition, the G-quadruplexes (10 μM) or G-triplexes (10 μM) solution were prepared in 10 mM Tris–HCl buffer (pH = 7.0) containing 0.5 mM Na_2_EDTA, 100 mM Na^+^ or 100 mM K^+^ or 100 mM Mg^2+^ or 100 mM Ca^2+^. Under molecular crowding conditions, additional 400 mL/L PEG 200 was added. The solution was heated to 95°C for 5 min, then cooled rapidly to 25°C and was allowed to incubate at 25°C for 30 min and overnight incubation at 4°C. After a sufficient mixing, the absorption signal at 295 nm (400 nm as control wavelength) was recorded at about 10°C. When the absorption signal became constant, the temperature was increased in steps of 1°C. At each temperature, the mixture was left to equilibrate for at least 1 min. Absorption signal was recorded when the signal did not change any more.

### Isothermal titration calorimetry

Isothermal titration calorimetry (ITC) measurements were performed using a MicroCalTM isothermal titration calorimeter iTC200 (GE Healthcare). DNA (50 μM) and Ca^2+^ (1 mM) solutions were both prepared in 10 mM Tris–HCl buffer (pH = 7.0). K^+^ (1 mM) solution was prepared in 10 mM Tris–HCl buffer (pH = 7.0) containing 0.5 mM Na_2_EDTA. All of the solutions were heated to 95°C for 5 min, then cooled rapidly to 25°C and were allowed to incubate at 25°C for 30 min and overnight incubation at 4°C. Then the ion (K^+^ or Ca^2+^, 1 mM) solution was titrated into the corresponding DNA (50 μM) solution. The titration included an initial injection of 0.4 μL ion solution followed by 19 injections of 2 μL ion solution every 120 s with stirring at 750 rpm at 16°C. To define the baseline, the ion was titrated into the same buffer without DNA under the same conditions. The titration data and binding plots after the baseline were subtracted were analyzed using MicroCal Origin software with the one-site binding model.

### Optical tweezers single-molecule assay

The DNA construct for single-molecule assay was prepared by sandwiching Hum15 (Table 1) between two double-stranded DNA (dsDNA) handles. One DNA handle (2028 bp) was labelled with biotin at the 5′-end, the other DNA handle (2690 bp) was labelled at the 3′-end by digoxigenin (Dig). The prepared DNA construct was incubated with anti-Dig antibody-coated polystyrene beads (diameter: 2.17 μm) in 10 mM Tris-HCl buffer (pH 7.0) for 1 h at room temperature to attach the DNA on the beads via the Dig/anti-Dig linkage. The beads attached with DNA construct and the beads coated with streptavidin (diameter: 1.87 μm) were injected into the top and bottom channels of a three-channel microfluidic chamber, respectively. The two types of beads were trapped by laser tweezers in the middle channel^43^, to which 10 mM Tris-HCl buffer (pH 7.0) containing different metal ions was injected. The two trapped beads were brought close to each other to tether the other end of the DNA construct to the streptavidin-coated bead via biotin/streptavidin linkage. Then, the steerable mirror of the laser tweezers that controls the anti-Dig-coated bead was moved away from the streptavidin-coated bead with a load rate of ~5.5 pN/s, and the force-extension (F-X) curves were recorded at 1000 Hz using a LabView program (National Instruments Corporation, Austin, TX). The secondary structure formed in the DNA molecule was unfolded when tension inside the tether was increased to a particular level. These raw data were filtered with a Savitzky-Golay function with a time constant of 10 ms using a Matlab program (The Math Works, Natick, MA). The change in contour length (Δ*L*) at a particular force (*F*) was calculated as the extension difference between the stretching and the relaxing traces at that force^44^.

## Supplementary Material

Supplementary Information Supporting information

## Figures and Tables

**Figure 1:**
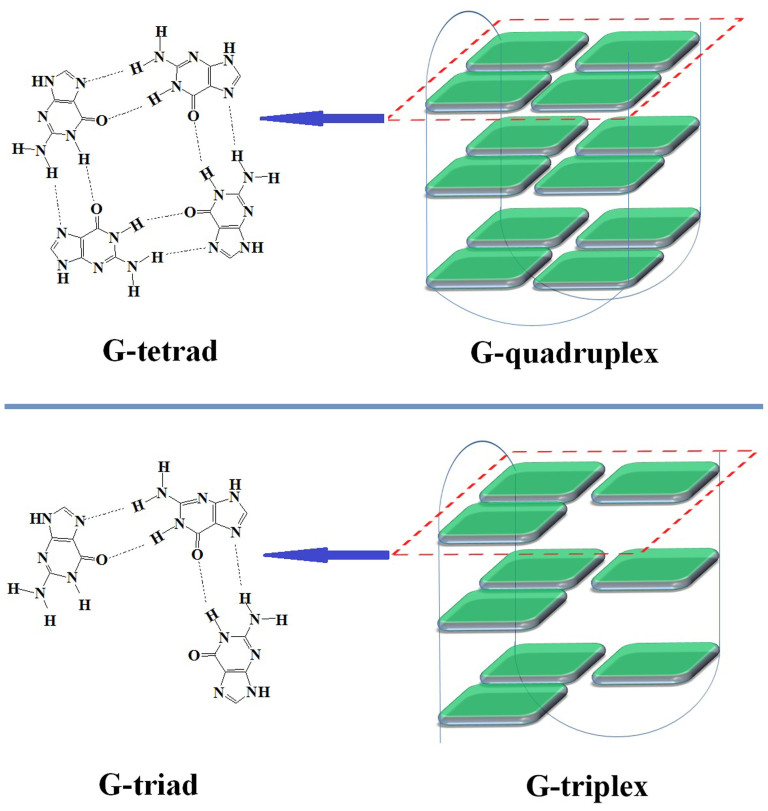
Schematic representation of G-tetrad, G-quadruplex, G-triad and G-triplex.

**Table 1:** G-rich oligonucleotides used in this work

DNAs	Sequence (from 5′ to 3′)^a^
TBA	GGTTGGTGTGGTTGG
TBA11	GGTTGGTGTGG
Hum21	GGGTTAGGGTTAGGGTTAGGG
Hum15	GGGTTAGGGTTAGGG
T_2_T_2_T_3_	GGGTTGGGTTGGGTTTGGG
T_2_T_2_	GGGTTGGGTTGGG
Hum15-1	GCGTTAGCGTTAGCG
Hum15-2	GTGTTAGTGTTAGTG
T_2_T_2_-1	GCGTTGCGTTGCG
T_2_T_2_-2	GTGTTGTGTTGTG
Hum9	GGGTTAGGG

^a^The underlines represent the G-tracts.

**Table 2:** Melting temperatures (*T*_m_) of the G-quadruplexes and G-triplexes under diluted or molecularly crowded conditions

	*T*_m_ of G-quadruplexes (°C)						*T*_m_ of G-triplexes (°C)					
	Hum21		T_2_T_2_T_3_		TBA		Hum15		T_2_T_2_		TBA11	
	Dilute	Crowded	Dilute	Crowded	Dilute	Crowded	Dilute	Crowded	Dilute	Crowded	Dilute	Crowded
Na^+^	58.4	63.0	44.0	46.4	22.7	23.8	32.6	43.5	29.6	33.0	Undetected	Undetected
K^+^	60.1	66.8	66.5	>80.0	48.9	51.1	43.2	49.9	51.5	56.3	26.4	30.0
Ca^2+^	54.9	56.3	53.8	54.4	30.3	29.0	47.7	52.0	59.0	64.5	24.2	34.3
Mg^2+^	39.4	43.3	31.8	40.9	21.2	27.6	31.2	46.0	40.9	48.9	20.7	24.2
