# Supplementary material for: Divalent cations and molecular crowding buffers stabilize G-triplex at physiologically relevant temperatures
Source: Sci Rep. 2015 Mar 19;5:9255. doi: 10.1038/srep09255 (PMC5380134; doi:10.1038/srep09255)
Supplement: Supplementary Information — Supporting information [file srep09255-s1.doc]

Supporting information for the article:

“**Divalent cations and molecular crowding buffers stabilize G-triplex at physiologically relevant temperatures**”

Hong-Xin Jiang, Yunxi Cui, Ting Zhao, Hai-Wei Fu, Deepak Koirala, Jibin Abraham Punnoose, De-Ming Kong* and Hanbin Mao*

1. **Structures of G-quadruplexes or G-triplexes under different conditions**

**Table S1.** Structures of G-quadruplexes or G-triplexes under different conditions

|  | | Dilute conditions | | | | Molecular crowding conditions | | | |
| --- | --- | --- | --- | --- | --- | --- | --- | --- | --- |
|  |  | Na^+^ | K^+^ | Ca^2+^ | Mg^2+^ | Na^+^ | K^+^ | Ca^2+^ | Mg^2+^ |
| G-  quadruplex | TBA | antiparallel | antiparallel | －－ | －－ | antiparallel | antiparallel | －－ | －－ |
|  | Hum21 | antiparallel | hybrid  (antiparallel dominant) | －－ | －－ | antiparallel | parallel | －－ | －－ |
|  | T_2_T_2_T_3_ | antiparallel | parallel | －－ | －－ | parallel | parallel | －－ | －－ |
| G-  triplex | TBA11 | －－ | antiparallel | －－ | －－ | －－ | antiparallel | －－ | －－ |
|  | Hum15 | hybrid  (antiparallel dominant) | hybrid  (antiparallel dominant) | parallel | parallel | antiparallel | hybrid  (parallel dominant) | parallel | parallel |
|  | T_2_T_2_ | hybrid  (parallel dominant) | hybrid  (parallel dominant) | parallel | parallel | parallel | parallel | parallel | parallel |

**2. Melting temperatures of G-quadruplexes or G-triplexes in the presence of different metal ions**

**Figure S1.** Melting temperatures of G-quadruplexes or G-triplexes in the presence of different metal ions under dilute condition or molecular crowding conditions.

**3. Temperature-absorbance profile of two G-rich oligonucleotides without G-triplex and G-quadruplex-forming potential**

**Figure S2.** Temperature-dependent absorption signal change (λ = 295 nm) for the two oligonucleotides without G-quadruplex and G-triplex-forming potential.

**4. Thermodynamic parameters for the formation of G-triplexes and G-quadruplexes**

**Table S2.** Thermodynamic parameters for the formation of G-triplexes and G-quadruplexes

| G-triplex or G-quadruplex | | Cation  (100 mM) | ∆*H*^ϴ^ (kJ/mol) | | ∆*S*^ϴ^ (J/mol•K) | | ∆G^ϴ^_37_ (kJ/mol) | |
| --- | --- | --- | --- | --- | --- | --- | --- | --- |
|  |  |  | Dilute | Crowded | Dilute | Crowded | Dilute | Crowded |
| G-triplex | TBA11 | Na^+^ | Undetected*^a^* | -178.6±4.6 | Undetected*^a^* | -602.0±15.3 | Undetected*^a^* | 8.32±0.19 |
|  |  | K^+^ | -184.9±4.1 | -125.7±3.6 | -616.3±13.8 | -414.8±12.0 | 6.47±0.18 | 3.05±0.13 |
|  |  | Ca^2+^ | -172.4±3.7 | -130.0±2.3 | -579.6±12.1 | -423.6±7.4 | 7.56±0.06 | 1.51±0.01 |
|  |  | Mg^2+^ | -308.8±9.8 | -315.4±9.9 | 1050.8±33.2 | 1062.5±31.4 | 17.51±0.55 | 14.54±0.51 |
|  | Hum15 | Na^+^ | -161.8±2.7 | -144.1±3.3 | -529.2±9.0 | -458.9±10.5 | 2.33±0.10 | -1.66±0.01 |
|  |  | K^+^ | -103.1±1.3 | -97.7±1.5 | -326.9±4.2 | -303.9±4.6 | -1.63±0.01 | -3.37±0.03 |
|  |  | Ca^2+^ | -113.6±3.6 | -80.7±0.8 | -356.0±11.5 | -248.3±2.6 | -3.05±0.07 | -3.64±0.02 |
|  |  | Mg^2+^ | -163.1±5.2 | -70.2±1.0 | -539.1±17.1 | -219.6±3.1 | 4.29±0.14 | -1.99±0.02 |
|  | T_2_T_2_ | Na^+^ | -178.2±3.8 | -178.5±4.7 | -589.5±12.7 | -585.2±15.3 | 4.89±0.09 | 3.22±0.04 |
|  |  | K^+^ | -99.3±2.7 | -75.9±0.8 | -306.4±8.5 | -230.9±2.4 | -4.12±0.08 | -4.21±0.02 |
|  |  | Ca^2+^ | -92.3±2.6 | -77.3±4.4 | -280.8±8.2 | -231.9±12.9 | -5.08±0.04 | -5.30±0.40 |
|  |  | Mg^2+^ | -154.9±6.5 | -83.8±0.47 | -498.3±21.0 | -261.4±1.5 | -0.22±0.04 | -2.62±0.02 |
| G-quadruplex | TBA | Na^+^ | -145.0±3.4 | -180.5±2.6 | -489.1±11.3 | -607.3±8.5 | 6.97±0.09 | 8.09±0.07 |
|  |  | K^+^ | -93.8±2.1 | -97.8±5.6 | -291.3±6.7 | -303.7±17.6 | -3.30±0.01 | -3.50±0.14 |
|  |  | Ca^2+^ | -101.3±1.5 | -95.3±1.4 | -333.1±4.8 | -315.1±4.6 | 2.13±0.01 | 2.58±0.01 |
|  |  | Mg^2+^ | -198.2±9.1 | -103.9±1.9 | -673.2±30.7 | -345.5±6.2 | 10.85±0.45 | 3.38±0.06 |
|  | Hum21 | Na^+^ | -173.3±16.8 | -65.4±1.8 | -542.5±56.1 | -191.1±5.5 | -4.87±0.58 | -5.79±0.05 |
|  |  | K^+^ | -168.0±9.2 | -81.2±1.0 | -523.8±30.7 | -239.1±3.0 | -5.34±0.30 | -6.91±0.05 |
|  |  | Ca^2+^ | -203.1±10.0 | -89.4±1.0 | -639.0±33.2 | -271.6±3.1 | -4.70±0.31 | -5.00±0.04 |
|  |  | Mg^2+^ | -131.6±2.4 | -92.9±1.5 | -442.0±7.8 | -294.8±4.7 | -0.56±0.01 | -1.37±0.01 |
|  | T_2_T_2_T_3_ | Na^+^ | -166.9±4.9 | -103.3±1.9 | -527.6±15.2 | -322.9±5.8 | -3.08±0.14 | -3.09±0.06 |
|  |  | K^+^ | -68.5±3.7 | Undetected*^b^* | -200.0±11.5 | Undetected*^b^* | -6.37±0.11 | Undetected*^b^* |
|  |  | Ca^2+^ | -102.4±1.6 | -110.5±2.1 | -314.8±5.1 | -339.5±6.5 | -4.69±0.05 | -5.08±0.04 |
|  |  | Mg^2+^ | -145.9±3.0 | -78.1±0.8 | -479.8±9.6 | -248.7±2.7 | 3.04±0.04 | -0.88±0.01 |

*^a^*Thermodynamic parameters cannot be calculated because no G-triplex is formed.

*^b^*Thermodynamic parameters cannot be calculated because the formed G-quadruplex is too stable to unfold in the tested temperature range.

**5. Effects of DNA concentration on the melting temperatures of G-triplexes**

**Figure S3.** Melting curves of the G-triplexes formed by TBA11 in the presence of different DNA concentrations under dilute conditions (**Left**) or molecular crowding conditions (**Right**).

**Figure S4.** Melting curves of the G-triplexes formed by Hum15 in the presence of different DNA concentrations under dilute conditions (**Left**) or molecular crowding conditions (**Right**).

**Figure S5.** Melting curves of the G-triplexes formed by T_2_T_2_ in the presence of different DNA concentrations under dilute conditions (**Left**) or molecular crowding conditions (**Right**).

**Table S3.** Effects of DNA concentration on the melting temperatures of G-triplexes under dilute or molecular crowding conditions

| Condition | G-triplex | | Cation | DNA concentration (μM) | | | |
| --- | --- | --- | --- | --- | --- | --- | --- |
|  |  |  |  | 7 | 10 | 12.5 | 15 |
| Under dilute conditions | TBA11 | K^+^ | | 26.6 | 26.4 | 26.4 | 26.4 |
|  |  | Ca^2+^ | | 25.6 | 25.0 | 25.5 | 25.5 |
|  |  | Mg^2+^ | | 21.0 | 20.7 | 21.3 | 20.5 |
|  | Hum15 | Na^+^ | | 32.1 | 32.6 | 32.5 | 32.3 |
|  |  | K^+^ | | 43.4 | 43.2 | 43.5 | 43.0 |
|  |  | Ca^2+^ | | 48.0 | 47.7 | 47.8 | 47.7 |
|  |  | Mg^2+^ | | 31.7 | 31.2 | 31.2 | 31.4 |
|  | T_2_T_2_ | Na^+^ | | 32.5 | 33.0 | 33.7 | 32.6 |
|  |  | K^+^ | | 51.2 | 51.5 | 50.8 | 51.4 |
|  |  | Ca^2+^ | | 59.4 | 59.0 | 59.4 | 59.4 |
|  |  | Mg^2+^ | | 41.1 | 40.9 | 39.3 | 39.8 |
| Under molecular crowding conditions | TBA11 | Na^+^ | | 23.6 | 23.6 | 24.4 | 24.3 |
|  |  | K^+^ | | 30.5 | 30.0 | 30.8 | 30.9 |
|  |  | Ca^2+^ | | 34.6 | 34.3 | 35.0 | 34.1 |
|  |  | Mg^2+^ | | 24.1 | 24.2 | 24.2 | 24.3 |
|  | Hum15 | Na^+^ | | 43.0 | 43.2 | 43.3 | 43.2 |
|  |  | K^+^ | | 50.0 | 49.9 | 50.8 | 50.4 |
|  |  | Ca^2+^ | | 51.2 | 52.0 | 51.5 | 50.5 |
|  |  | Mg^2+^ | | 44.7 | 46.0 | 46.8 | 45.5 |
|  | T_2_T_2_ | Na^+^ | | 30.1 | 29.6 | 29.5 | 30.1 |
|  |  | K^+^ | | 55.3 | 56.3 | 55.1 | 56.0 |
|  |  | Ca^2+^ | | 64.9 | 64.5 | 64.1 | 65.3 |
|  |  | Mg^2+^ | | 51.1 | 48.9 | 48.4 | 49.9 |

**6. The intermolecular G-quadruplex formed by Hum9**

**Figure S6.** CD spectra of Hum9 in the presence of different metal ions under dilute (**Top**) or molecular crowding conditions (**Middle**). Black trace represents the CD spectra of Hum15 in the presence of 100 mM Ca^2+^. The melting temperature of the intermolecular G-quadurplex formed by Hum9 in the presence of K^+^ was shown in the **Bottom** panel.

**7. The effects of Ca^2+^ concentration on the *T*_m_ of G-triplex formed by Hum15**

**Table S4**. Melting temperature of the G-triplexes formed by Hum15 in the presence of different Ca^2+^ concentrations

| Condition | Ca^2+^ concentration (μM) | | | | | | |
| --- | --- | --- | --- | --- | --- | --- | --- |
|  | 0 | 2 | 5 | 10 | 20 | 50 | 100 |
| Dilute | -- | 35.0 | 37.8 | 39.7 | 42.9 | 45.5 | 47.8 |
| Crowding | 25.9 | 40.8 | 43.1 | 45.6 | 48.0 | 50.3 | 52.3 |

**8. Optical tweezers single-molecule assay for Hum15**

**8.1 Under dilute conditions**

**8.1.1 In the presence of 100 mM K^+^**

**Figure S7.** Change in contour length (∆L) (**a**) and rupture force (**b**) histograms for structures in Hum15 in the prescence of 100 mM K^+^ in a 10 mM Tris buffer (pH 7.0). The black solid curves in these two figures are Gaussian fittings.

**8.1.2 In the presence of 100 mM K^+^ + 2 mM Ca^2+^**

**Figuee S8.** Change in contour length (∆L) (**a**) and rupture force (**b**) histograms for structures in Hum15 in the prescence of 100 mM K^+^ and 2 mM Ca^2+^ in a 10 mM Tris buffer (pH 7.0). The black solid curves in these two figures are Gaussian fittings.

**8.2 Under molecular crowding conditions**

**8.2.1 In the presence of 100 mM K^+^**

**Figure S9.** Change in contour length (∆L) (**a**) and rupture force (**b**) histograms for structures in Hum15 in the prescence of 100 mM K^+^ and 40% DMSO in a 10 mM Tris buffer (pH 7.0). The black solid curves in these two figures are Gaussian fittings.

**8.2.2 In the presence of 100 mM K^+^ + 2 mM Ca^2+^**

**Figure S10.** Change in contour length (∆L) (**a**) and rupture force (**b**) histograms for structures in Hum15 in the prescence of 100 mM K^+^, 2 mM Ca^2+^ and 40% DMSO in a 10 mM Tris buffer (pH 7.0). The black solid curves in these two figures are Gaussian fittings.
